# Reducing pancreatic fistula after left-sided pancreatectomy—the search continues

**DOI:** 10.1093/bjs/znaf033

**Published:** 2025-02-25

**Authors:** Dyre Kleive

**Affiliations:** Department of HPB Surgery, Oslo University Hospital, Rikshospitalet, Oslo, Norway

Postoperative pancreatic fistula is the main cause of morbidity and readmissions after left-sided pancreatectomy^1,2^. Although the overall burden of complication caused by fistula is less severe in left-sided resections than after pancreatoduodenectomy, it remains a considerable and unsolved problem. For patients, a postoperative pancreatic fistula represents an exhausting and tiring consequence at best. For clinicians, it is equally frustrating and time-consuming. For healthcare systems, it is costly. Within well-designed controlled trials the rate of clinically relevant postoperative pancreatic fistulae (grades B/C, as defined by the International Study Group of Pancreatic Surgery (ISGPS)) is as high as 30%^3^. The cost of clinically relevant postoperative pancreatic fistula after left-sided pancreatectomy is high^4^. As such, numerous attempts to avoid and reduce the occurrence of clinically relevant postoperative pancreatic fistula have been made, with no universal and few lasting successes. With the increased use of minimally invasive techniques, the pancreas is frequently divided with a stapler. The anatomical site of transection in the neck, body or tail of the pancreas may be related to risk of fistulae, but a standardized definition of left-sided pancreatectomy has only recently been proposed^5^, hence limiting comparisons based on prior studies. In the setting of several options and variation in management, one can imagine all existent personal beliefs and different convictions between surgeons: use of different type of stapler size, with or without enforced staple lines; use of different type of drains at the resection site; or, no drains in some and selective use of drains in others; variations in how to manage the pancreatic remnant—should it be covered with haemostatic material or not? Is there a specific type of suture of the transection line or of the pancreatic duct that may surely close the duct for good to avoid a leak? Although several of these techniques have been explored in single- and multicentre retrospective studies, the data derived from high-level evidence such as in RCTs have been few and far between.

In this issue, Sumiyoshi and co-authors present their findings from a multicentre RCT investigating the effect of pre-compression *versus* non-compression of the pancreatic gland before transection of the parenchyma during left-sided pancreatectomy^6^. Rate of clinically relevant postoperative pancreatic fistula was the chosen primary endpoint for the trial. In the intervention group, the pancreas was compressed with a straight 70-mm intestinal bulldog clip (from AESCULAP^®^ Bulldog Clips; B. Braun, Germany) for 10 min before pancreatic parenchyma transection. Transection of the pancreas was performed using a reinforced Tri-Staple™ (Covidien) with two size options (purple or black) at the discretion of the surgeon. Importantly, randomization was stratified by the thickness of pancreatic parenchyma, surgical approach, and institution. No somatostatin analogues were given intraoperatively or post surgery and all patients received at least one drain. Overall, clinically relevant postoperative pancreatic fistula was observed in 12.9% with no significant difference between the two groups. A subgroup analysis of patients with BMI greater than 25 revealed a significant difference in clinically relevant postoperative pancreatic fistula in favour of the pre-compression group (0/19 patients for pre-compression *versus* 5/17 patients for no compression respectively).

The authors are to be commended for conducting a trial that aligns well with real-world practice. Furthermore, a recent guideline on minimally invasive pancreatic surgery gives a strong recommendation based on a low level of evidence that gradual stepwise compression is advised, highlighting the need for this trial^7^. However, some caution should be taken when interpreting the results. The sample size is limited and, thus, the claim of equivalence between the two groups can always be questioned in a study designed as a superiority trial. Considering this, the subgroup results of patients with obesity (defined as BMI > 25) needs further investigation. Lastly, some pancreatic glands have a very hard parenchyma due to fibrotic or inflamed tissue. Not even manual compression will have the slightest impact on reducing thickness at the transection site. Nevertheless, this study sheds important light to a technical feature most likely used (in various forms) by pancreatic surgeons around the world. If anything, the results of this trial challenges the personal belief or conviction of a technical solution to avoid clinically relevant postoperative pancreatic fistula—at least for the moment.

Fortunately, more insights into the risk factors for clinically relevant postoperative pancreatic fistula have been gained in the recent years. Importantly, two risk calculators for postoperative pancreatic fistula after left-sided pancreatectomy have been published^8,9^. More are likely to come. Among some established risk factors are coexisting diabetes, pancreatic thickness at the transection site, and pancreatic duct diameter. These factors may allow for stratification. A recent trial investigating the non-inferiority of drain omission after distal pancreatectomy used such a stratification^10^. Although drain omission was found to be non-inferior to drain placement in the overall cohort, the study also showed that a no-drain policy reduced the risk of major morbidity and clinically relevant postoperative pancreatic fistula in patients with a low risk of pancreatic fistula. Hopefully, this high-level evidence will be sufficient for pancreatic surgeons to omit drains, but translation of findings in trials and their impact on practice change is another issue. Importantly, stratification according to risk also allows for multimodal interventions in high-risk individuals, which could serve as a future possible strategy to reduce the rate of clinically relevant postoperative pancreatic fistula.

Lastly, all evidence must be considered in light of how an endpoint is defined. The understanding of postoperative pancreatic fistulae has evolved during the last decades^1,2^. Although the updated ISGPS definition for clinically relevant postoperative pancreatic fistula is now widely accepted, new insights in the clinical burden and costs of grade B fistulae have been established. Based on the need for either prolonged drainage, pharmacological treatment, or interventional procedures, there seems to be an incremental cost and worse clinical outcome^11^. Although this type of knowledge is useful, leakage prevails at a fairly constant rate. Consequently, it is safe to say that although new definitions with more granular descriptions have emerged, the problem has not been solved. In an era where novel single- and composite endpoints are flourishing, it can be hard to keep the eye on the ball. The search for that something that might work will surely continue. Hopefully the search and investigation will be in the shapes and forms like the current study provided by Sumiyoshi and colleagues. For now, the result of the current trial at least reminds us that a firmly held opinion is nothing more than a qualified guess until high-level evidence is available.

## Data Availability

Not applicable

## References

[1] Bassi C, Dervenis C, Butturini G, Fingerhut A, Yeo C, Izbicki J et al Postoperative pancreatic fistula: an international study group (ISGPF) definition. Surgery 2005;138:8–13 16003309 10.1016/j.surg.2005.05.001

[2] Bassi C, Marchegiani G, Dervenis C, Sarr M, Abu Hilal M, Adham M et al The 2016 update of the international study group (ISGPS) definition and grading of postoperative pancreatic fistula: 11 years after. Surgery 2017;161:584–591 28040257 10.1016/j.surg.2016.11.014

[3] de Rooij T, van Hilst J, van Santvoort H, Boerma D, van den Boezem P, Daams F et al Minimally invasive *versus* open distal pancreatectomy (LEOPARD): a multicenter patient-blinded randomized controlled trial. Ann Surg 2019;269:2–9 30080726 10.1097/SLA.0000000000002979

[4] van Bodegraven EA, Francken MFG, Verkoulen K, Hilal A, Dijkgraaf M, Besselink MGW et al Costs of complications following distal pancreatectomy: a systematic review. HPB (Oxford) 2023;25:1145–1150 37391314 10.1016/j.hpb.2023.03.007

[5] van Ramshorst TME, van Hilst J, Boggi U, Dokmak S, Edwin B, Keck T et al Standardizing definitions and terminology of left-sided pancreatic resections through an international Delphi consensus. Br J Surg 2024;111:znae039 10.1093/bjs/znae039 38686655

[6] Sumiyoshi T, Uemura K, Seo S, Fujii T, Satoi S, Miwa T et al Impact of pre-compression vs. non-compression before parenchyma transection in left-sided pancreatic resection on clinically-relevant pancreatic fistula rate: a multicenter, randomized controlled trial. Br J Surg 2025. in press 10.1093/bjs/znaf008 40036743

[7] Abu Hilal M, van Ramshorst TME, Boggi U, Dokmak S, Edwin B, Keck T et al The Brescia internationally validated European guidelines on minimally invasive pancreatic surgery (EGUMIPS). Ann Surg 2024;279:45–57 37450702 10.1097/SLA.0000000000006006 PMC10727198

[8] Bonsdorff A, Ghorbani P, Helanterä I, Tarvainen T, Kontio T, Belfrage H et al Development and external validation of DISPAIR fistula risk score for clinically relevant postoperative pancreatic fistula risk after distal pancreatectomy. Br J Surg 2022;109:1131–1139 35983583 10.1093/bjs/znac266 PMC10364701

[9] De Pastena M, van Bodegraven EA, Mungroop TH, Vissers FL, Jones LR, Marchegiani G et al Distal pancreatectomy fistula risk score (D-FRS): development and international validation. Ann Surg 2023;277:e1099–e1105 35797608 10.1097/SLA.0000000000005497

[10] van Bodegraven EA, Balduzzi A, van Ramshorst TME, Malleo G, Vissers FL, van Hilst J et al Prophylactic abdominal drainage after distal pancreatectomy (PANDORINA): an international, multicentre, open-label, randomised controlled, non-inferiority trial. Lancet Gastroenterol Hepatol 2024;9:438–447 38499019 10.1016/S2468-1253(24)00037-2

[11] Maggino L, Malleo G, Bassi C, Allegrini V, McMillan MT, Borin A et al Decoding grade B pancreatic fistula: a clinical and economical analysis and subclassification proposal. Ann Surg 2019;269:1146–1153 31082914 10.1097/SLA.0000000000002673

